# The experiences of adjuvant endocrine therapy for women breast cancer survivors: A literature review

**DOI:** 10.1097/MD.0000000000036704

**Published:** 2023-12-22

**Authors:** Yaoyao Long, Shaoju Xie, Qinghua Liu, Fan Xu, Qiao Li, Na Wang, Youcai Zhang

**Affiliations:** a Department of Oncology, Deyang people’s hospital, Deyang, China.

**Keywords:** AET, breast cancer, experiences, literature review

## Abstract

**Introduction:**

Adjuvant endocrine therapy (AET) is commonly recommended for non-metastatic breast cancer survivors. However, the side-effects associated with AET can have a negative impact on survivors’ functional status and quality of life. Understanding the factors influencing adherence to AET is crucial in improving its utilization among female breast cancer survivors.

**Conclusions:**

This literature review critically evaluated 15 articles to explore the experiences of female breast cancer survivors in adhering to and persisting with AET. The findings highlight that while AET can cause drug side-effects, the involvement of healthcare professionals (HCP) plays a significant role in facilitating better use of AET. Unfortunately, many HCP fail to discuss vital information related to AET or provide guidance on managing side-effects and daily medication. Consequently, survivors often lack guidance in these areas. Despite experiencing discomfort, survivors maintain a positive attitude towards using AET and employ self-management strategies and social networks to overcome barriers. The impact of HCP on AET adherence among female breast cancer survivors is substantial. Future research should focus on understanding perspectives that promote HCP involvement, which will inform practical intervention strategies in clinical practice.

## 1. Introduction

Breast cancer is the most common cancer in women all over the world and the second leading cause of cancer death.^[1]^ The majority of non-metastatic breast cancer survivors undergo adjuvant endocrine therapy (AET).^[2]^ Guidelines recommend an extension of AET to 10 years in high-risk female breast cancer survivors.^[3]^ This is a huge challenge for female breast cancer survivors who must take AET every day.^[4,5]^ AET has always been the standard of treatment for breast cancer survivors to prevent possible recurrence in women with hormone receptor-positive breast cancer,^[4]^ including aromatase inhibitors (anastrozole, letrozole, and exemestane) that inhibit estrogen synthesis or selective estrogen receptor modulators (SERMs) (tamoxifen).^[6]^ However, breast cancer patients receiving any type of AET may experience drug side-effects, and the multiple symptoms associated with the side-effects have a negative impact on the survivor functional status and quality of life, leading to changes in the survivor medication-taking behavior (MTB).^[7–9]^ MTB can be divided into 2 categories: persistence (continuing the treatment for the prescribed period) and adherence (acting in accordance with the prescribed interval and dosage of the treatment).^[10]^ Previous studies demonstrated that women with low MTB to AET had an increased risk of mortality, an increased recurrence rate of patients^[11,12]^ and increased medical costs.^[13]^ Although most survivors showed a positive attitude toward taking AET, they did not perform well in terms of adherence and persistence.^[14,15]^ Therefore, it is necessary to understand what female breast cancer survivors experience with AET and what interventions can promote MTB. This will help female breast cancer survivors improve adherence and persistence in taking medications, promote long-term self-management, improve quality of life, and ultimately improve survival.

## 2. Search strategy

As a result, this review of the literature explores how female breast cancer survivors feel about AET what factors hinder adherence and persistence in taking AET and what interventions can promote MTB.

Following the Preferred Reporting Items for Systemic Reviews and Meta-Analyses (PRISMA)^[16]^ guidelines (Appendix 1, Supplemental Digital Content, http://links.lww.com/MD/L126), 6 databases were searched including CINAHL, MEDLINE, Psych Articles, PubMed, Cochrane Library, and Academic Search Complete. The search strategy (Appendix 2, Supplemental Digital Content, http://links.lww.com/MD/L127), based mainly on Medical Subject Headings (MESH) terms were “breast cancer ” OR “breast cancer survivors” OR “breast cancer patients” OR “breast cancer clients” AND “experiences” OR “perceptions” OR “attitudes” OR “views” OR “feelings” OR “perspective” OR “opinion ”OR “qualitative ” AND “ adjuvant endocrine therapy” OR “endocrine therapy” OR “AET” OR “ET” OR “tamoxifen” OR “aromatase inhibitors” OR “drug therapy” OR “hormone therapy.”

The limits used were: the English language, articles from the year 2012 to 2022, peer-reviewed, journal articles, and the article describing the experiences of AET for women breast cancer survivors or different interventions which can promote MTB.

## 3. Results

As for data extraction, firstly, after a thorough search of all keywords, the record totaled 686. Secondly, 7 records were identified through a hand search of the reference list searches of articles. Thirdly, after duplicates were removed, 356 records remained, 306 of which were excluded based on the title abstract. Fourthly, systematic literature reviews were removed, which left the remaining 20 records. After articles were scrutinized more closely, 5 records were excluded: Unpublished conferences (n = 2) and including men breast cancer survivors (n = 3). As a result, only fifteen articles (Appendix 3, Supplemental Digital Content, http://links.lww.com/MD/L128) met the inclusion criteria: eleven qualitative studies, 1 RCT study, 1 longitudinal study, 1 randomized controlled feasibility trial, and 1 observational study. Additionally, participants came from Canada, America, England, Brazil, and Ireland. Sample sizes ranged from 12 to 921.

Following a review of 15 articles, 3 themes emerged: factors hindering adherence and persistence in taking AET, feelings about using AET, and coping strategies used to improve MTB. Each topic will now be described.

### 3.1. Factors hindering adherence and persistence in taking AET

MTB included adherence and persistence, which are 2 different terms. Non-adherence and non-persistence to AET were influenced by many factors. While some of the factors that hindered AET adherence and persistence were similar, there were still some differences. Hence, this review will discuss the factors hindering different MTBs separately.

Adherence was defined as a level of obedience to the prescribed treatment or medication in terms of timing, dosage, and frequency.^[17]^ Female breast cancer survivors had a high level of adherence.^[5]^ Liu et al^[14]^ and Wickersham, Happ and Bender^[7]^ reported overall adherence up to 88% and 87.8%, respectively. However, non-adherence rates rose significantly over time. Moon et al^[15]^ showed that 37 to 48% of survivors were non-adherent after 1 year. The literature review found that many factors hindered adherence, including drugs side-effects, quality of life declines, perception of the risk of recurrence, women beliefs, inadequate medication management techniques, lack of symptom management information from the healthcare provider, general distrust in medication, lack of knowledge and conviction in the efficacy of the treatment, economic cost, and lack of social support. More than half of the studies identified drug side-effects and decreased quality of life as the top barriers to adherence.^[4,5,8,9,18,19]^ In a study, participants reported 3 to 6 side-effects, and 10 participants reported 5 or more side-effects.^[7]^ A variety of symptoms can result from side-effects, including vasomotor symptoms, sexual dysfunction, insomnia, fatigue, cognitive dysfunction, pain, functional limitations, mood disorders, anxiety,^[5]^ and weight gain.^[18]^ More than a third described the profound effects these side-effects have on their daily lives.^[4]^ Interestingly, in another study, some participants reported that the side-effects subsided as they continued to take the AET medication.^[20]^ Jacobs et al^[19]^ reported that low-adherence survivors’ fears of medication, conflicting attitudes, and negative beliefs were ubiquitous. They thought the benefits of treatment outweighed the side-effects.^[8]^ When some survivors reached a balance point they would weigh the pros and cons of continuing to use AET^[5]^ because some survivors argued that reducing the risk of recurrence was not worth sacrificing the quality of life. Some studies claimed that women with low-adherence had a negative view of AET and generally distrust prescription drugs.^[21]^ Conversely, eleven women who undoubtedly took AET were more compliant because they believed in the value and importance of the drug.^[7]^ Harrow et al^[4]^ also evidenced that severe side-effects in these women did not necessarily affect their adherence. Survivors reported failure to manage AET-related symptoms, leading to low-adherence.^[5]^ They lacked symptom self-management skills and information and mentioned that healthcare providers had not discussed AET-related information or provided symptom management information.^[8,21]^ Wickersham, Happ and Bender^[7]^ reported almost none of the 12 participants received guidance on medication use, side-effects, and daily medication management. While some survivors sought social support, emotional support from friends and family was minimal and no contact was maintained with other survivors.^[19]^ A study also reported that some people missed taking the medication without realizing the potential consequences.^[4]^ A small number of survivors said they had difficulty paying for medications^[18]^ and no health insurance,^[14]^ leading to reduced adherence.

Furthermore, Moon et al^[15]^ reported that age, ethnic minority groups, being employed, and women who have higher levels of distress, may be at higher risk of non-adherence. However, Liu et al^[14]^ reported that low levels of education, put women at higher risk of non-adherence, but age was no significant correlation. Due to the source and selection of the sample size, this conclusion might potentially have bias.

Persistence was defined as the continuation of treatment from its inception to its termination.^[17]^ The main reason for hindering the persistence of AET was also the side-effects.^[9,22]^ A small percentage of women experience worsened side-effects overtime or find their symptoms unbearable, forcing them to discontinue AET.^[7,8]^ Hurtado-de-Mendoza et al^[9]^ described 2 participants stopped AET use. For non-persistent women, their awareness of the risks associated with breast cancer recurrence declined later in treatment. After 3 years, 4 (18%) women in a study sample discontinued treatment.^[22]^ Cahir et al^[21]^ demonstrated that non-persistence was linked to a strong distrust of medication and the health-care system, a lack of perceived need for treatment, and a preference for a good quality of life with little concern or thought given to future outcomes. Non-persistent women perceived a lack of continuity of care and inadequate personal attention.^[21]^ They wanted to be more involved in treatment decisions and get information on AET. Jacobs et al^[19]^ also suggested that social support could reduce the risk of AET termination. Moreover, Humphries et al^[23]^ demonstrated that not establishing a routine was a major obstacle for non-persistent women.

Overall, MTB in female breast cancer survivors was positive. However, due to side-effects and other obstructive factors, leading to adherence and persistence were reduced over time, which seriously affected the survival quality and expected effects of survivors.

### 3.2. Feelings about using AET

Initially, survivors were happy to accept the advice of a healthcare professional (HCP) to take AET,^[4,8]^ like a doctor advice. It was not seen as an option, but as a necessary next step to save lives.^[5]^ Some survivors thought the use of AET was a precautionary measure, which made them feel like they were actively doing something to prevent cancer from coming back and made them feel like they were still alive.^[8]^ Other survivors thought it was part of the ongoing treatment and they had gone through all the other parts, so there was no reason to refuse to take AET.^[4]^ Most survivors recognized the value of taking AET^[7]^ and described a strong motivation to take AET to reduce or block estrogen to prevent breast cancer recurrence, but they didn’t have insight into their medications (AET) and how or why estrogen blocking or reduction translates into a reduced risk of recurrence.^[19]^ Survivors even did not ask too much about the risks associated with AET, which they felt were not at the forefront of the discussion. If taking AET meant they could live and stop cancer from coming back, a survivor indicated she was willing to experience some side-effects.^[8]^ This may seem like a small price to pay for not having breast cancer anymore.

Over time, however, this positive attitude showed signs of shifting to a negative one, because survivors taking AET to produce a range of persistent symptoms that could cause their quality of life and mental state to be strongly affected. Survivors mentioned experiencing unexpected and incomprehensible symptoms.^[5]^ Survivors generally reported 3 types of side-effects.^[19]^

Firstly, physical appearance and menopausal symptoms (e.g., weight gain, hair loss, body image, hot flashes, and vaginal dryness), changed women sense of identity. Secondly, daily functioning (e.g., pain, fatigue, sleep difficulties, sexual functioning) led to poor memory, poor concentration at work, sweating interfering with sleep, and not having enough energy to participate in life and work. Thirdly, emotional well-being(e.g., mood fluctuations, depressed mood, anxiety). Moon et al^[8]^ reported 2 women experiencing severe depression and suicidal thoughts. Survivors suffered from these symptoms and were unable to tell friends or families about it because they may not be able to understand the pain that survivors experience.^[5]^ Survivors lacked relevant information about the AET, had no idea there would be such a reaction, and even received contradictory advice from HCP.^[7]^ For instance, when pain occurred, the person being consulted did not know whether it was the cause of the disease or the reason for taking AET. Furthermore, there was also a lack of help from HCP to manage these symptoms, leading to the failure to self-manage symptoms.^[8]^ Although Farias et al^[20]^ reported women received effective information about AET from HCP and HCP was involved in the management of their AET treatment, respondents included only women who were highly educated and better paid. All of the foregoing contributed to survivors’ ambivalence, as they recognized that AET could improve survival but did not deal with the side-effects and how they affected their daily lives.^[9,19]^ Survivors gradually realized that taking AET was an option. Survivors began to doubt their original beliefs and think about the pros and cons of continuing to take AET,^[5,22]^ which was also a major turning point in whether survivors continued to take AET. In the study,^[8]^ survivors who had discontinued their medications expressed they were willing to risk relapse to improve their quality of life rather than focus on the length of their lives and they believed they made the right choice. Similarly, in another study 3 women deliberately did not comply for a short period, suspended treatment due to the side-effects they were experiencing, and did so without seeking the advice of the HCP.^[4]^ However, based on professional advice on restoring endocrine therapy, they appeared to be refactoring their belief in the necessity of endocrine therapy. It is worth mentioning that more than 2-thirds of women were keen to continue treatment and were battling side-effects.^[7,8]^

### 3.3. Using a variety of coping strategies to improve MTB

According to this literature review, there is a strong need for effective support strategies for female breast cancer survivors to improve MTB and help survivors with poor MTB overcome various barriers to AET use. This requires not only medication self-administration strategies, but also a supportive social network to help and support their continued use of AET. The following are specific interventions:

At first, when prescribing AET, survivors need comprehensive information about the side-effects associated with the prescribed medication and the likelihood of these side-effects occurring. For example, joint pain may arise with the use of AET and could prevent them from doing housework, driving, cooking, etc. One survivor described the benefits of knowing the side-effects, which allowed her to organize her day more rationally.^[18]^ Strategies to deal with side-effects are available through formal resources (e.g., brochures, online resources, components of group education sessions) or reach out to through tumor survival programs, primary care providers, and peer support groups. Interventions such as exercise plans,^[24]^ hypnosis, yoga, and relaxation strategies may help women better cope with long-term AET use.

Secondly, HCP should provide information on the negative consequences of poor MTB, especially the risk of breast cancer recurrence associated with not taking the drug, compared to taking the drug as prescribed. It could help survivors develop a firm belief in the importance of AET. When survivors held positive beliefs about drugs, their high perceptions of relapse risk outweighed concerns about long-term medication use and help survivors deal with side-effects by making lifestyle changes and committing to a positive mindset.^[22]^

Thirdly, ongoing assessment and management during AET use in survivors. Although new evidence supporting 10 years of AET use, suggested that any intervention to improve adherence would need to be in the community, rather than in secondary care settings, there was little evidence of consistent, routine monitoring of medication in the community or follow-up clinics.^[4]^ This study recommended the establishment of a new community-based service model to facilitate long-term medication self-management in survivors. Fourthly, strengthening positive interactions between survivors and doctors, as well as other social interactions. A survey of low-income female breast cancer survivors found that patient-centered communication and targeted interventions for treatment side-effects could improve AET adherence among low-income, underserved survivors.^[14]^ Farias et al^[20]^ demonstrated the importance of enhanced communication with physicians among better-paid survivors and reported on 4 main functions of physician-patient communication: information exchange, taking and continuing decisions on AET, enabling patients to self-manage and monitor for potential side-effects, and emotional support. Physicians could exchange information with patients, so they understand and improve patient health literacy, understanding the benefits and knowledge of AET. Patients are given the authority to make decisions about their own care by their doctors. Patients expressed trust and confidence in their doctor, which aided them in seeking care when it was needed. Patients reported a high level of self-efficacy in managing AET on their own. In addition to the doctor, the support of other breast cancer survivors, friends, or family members could have a significant impact on survivors’ beliefs about the importance of AET, thereby improving MTB.^[19,22,23]^ Jacobs et al^[19]^ recommend group meeting interventions for survivors during the first few years of AET to gain support and the ability to share and learn from the experiences of others. Survivors preferred to use virtual video platforms at home rather than coming to the hospital. Survivors considered this period a difficult adjustment. Fifthly, reducing drug costs. One participant mentioned that the drug was not cheap, it was expensive.^[18]^ It is recommended to provide more medical security services, especially for low-income groups.

Sixthly, survivors could establish a medication schedule, such as placing antihormonal medications in prominent locations, taking antihormonal medications with other medications, and using pill boxes.^[18]^ This works especially well for survivors who unconsciously forget to take their medicine. In a pilot randomized controlled trial, Graetz et al^[25]^ tested the use of an app designed with or without weekly reminders for participants to report real-time symptoms and AI outside of clinical visits in survivor use and built-in alert messages for participants to be emailed to the nursing team. Both participants and providers participating in the study expressed positive experiences and reported higher adherence to AET use at week 8. However, the app was only tracked for 8 weeks, and future research testing should include the entire treatment period to confirm the App effectiveness.

## 4. Methodology critique

As we all know, when planning a study, researchers must consider the philosophical worldview assumptions that they bring to the study, the strategy of inquiry related to this worldview, and the specific methods or procedures of research that translate the approach into practice.^[26]^ Regarding the experience of AET in female breast cancer survivors, the worldviews included post-positivism, positivism, and interpretivism in 15 studies. Positivism is considered a scientific method based on rationalism, only when researchers systematically and dispassionately observe the data of the empirical world can researchers discover legitimate patterns with evidence, for example, Wickersham, Happ and Bender^[7]^ and Hurtado-de-Mendoza et al^[9]^ Post-positivism reflects the need to identify and assess the causes of impact from research questions and allows for complexity and personalization of patient care, builds on knowledge as evidence-based practices change, and helps develop usable and transferable knowledge for those providing care,^[27]^ like Graetz et al^[25]^ and Paulo et al^[24]^ The individual perception of social reality in the process of interaction between consciousness and object, according to Interpretivism, was socially constructed, for instance, Lambert et al^[22]^

Fifteen studies (eleven qualitative and 4 quantitative) were included in this review. On the one hand, 4 quantitative studies included different designs. Paulo et al^[24]^ used a randomized controlled trial study. Through simple random sampling, control and intervention groups were identified, providing exercise plan intervention for female breast cancer survivors. While this approach was better at preventing the effects of human factors and validating the findings, the study lacked physical function tests, small sample sizes, and self-reported assessments of questionnaires that were often inferior to objective measurements. Moon et al^[15]^ used a longitudinal study which was also known as follow-up study and observed the dynamics of the variables over a 1-year follow-up period, showing the chronological order between the independent and dependent variables. Its results were more convincing than the results of the cross-section study, but the racial differences in the study sample were small. Liu et al^[14]^ used an observational study that assessed the effect of doctor-patient communication on adherence 3 years after diagnosis by non-randomizing comparisons of specific low-income breast cancer women who used initiated AET. However, the study sample did not represent more racial differences and measured results by patient self-reporting, so the reported high adherence rate may be affected by bias in social expectation responses. Graetz et al^[25]^ used a randomized controlled feasibility trial, since it was a small pilot trial, statistically, significant differences in the findings could not be detected, and the samples came from a single clinic, which limited the generalizability of the results. Nevertheless, using this approach, the necessary revisions and refinements of the intervention programme can be made based on the results of the study.

On the other hand, the remaining eleven qualitative studies provided the opportunity to understand the perception or experience of using AET from a patient perspective. Qualitative research is suitable for the detailed and dynamic description and analysis of individual things, and the exploration of special phenomena. It can offer new perspectives on the problem, such as Lambert et al^[22]^ investigated how personal, social, and structural factors shape women AET experiences using a relational autonomy lens, and Humphries et al^[23]^ used the Theory of Planned Behavior to explore women experience with AET. Its limits included purposeful sampling, and the sample size was generally small and not universal.^[4,5]^ These qualitative research studies collected data through various interview methods and engaged in purposeful conversations with participants to gain a comprehensive and in-depth understanding of the participant experiences, but participants may have experience recall bias.^[8,22]^

Despite the fact that the search strategy aimed to include all relevant literature, it was only limited to English language publications. It may miss articles that add value to the comment. Furthermore, many of the studies included in the review failed to distinguish between aromatase inhibitors and tamoxifen.

## 5. Conclusion

This literature review explored the experience of female breast cancer survivors of adherence and persistence to using AET. Through the rigorous procedure of screening studies and comprehensive analysis of selected studies, many positive results and findings have been obtained. The results of this literature review showed that most survivors experienced discomfort with AET use, but they still had a positive attitude towards using AET, and when they encountered various barriers to using AET, they could promote MTB through self-management strategies and supportive social networks. In addition, HCP had a particular impact on MTB during AET in female breast cancer survivors and was very important. In the future, more research could focus on understanding the perspectives that support female breast cancer survivors embracing HCP using AET, which will help inform practical intervention strategies in clinical practice.

## Acknowledgments

The author thanks Dr Catherine Browne, for her guidance and advice. In addition, the author thanks the librarians at the Munster Technological University, Ireland, for their kind assistance during the retrieval phase.

## Author contributions

**Conceptualization:** Yaoyao Long, Fan Xu.

**Data curation:** Qiao Li, Na Wang.

**Methodology:** Fan Xu, Youcai Zhang.

**Software:** Qiao Li, Na Wang.

**Supervision:** Qinghua Liu.

**Validation:** Yaoyao Long, Fan Xu.

**Visualization:** Yaoyao Long.

**Writing – original draft:** Yaoyao Long, Shaoju Xie, Fan Xu.

**Writing – review & editing:** Qinghua Liu.

## Supplementary Material






