# Supplementary material for: The experiences of adjuvant endocrine therapy for women breast cancer survivors: A literature review
Source: Medicine (Baltimore). 2023 Dec 22;102(51):e36704. doi: 10.1097/MD.0000000000036704 (PMC10735117; doi:10.1097/MD.0000000000036704)
Supplement: Supplementary file 1 [file medi-102-e36704-s001.docx]

## Adapted PRISMA 2009 Flow Diagram

Additional records identified through other sources

(n=7)

Records identified through database searching
(n = 686)

Records after duplicates removed
(n = 356)

Records excluded
(n =30)

Full-text articles excluded, with reasons
(n = 5)

Unpublished conference(n=2) and Including men breast cancer survivors(n=3)

Studies included in LR
(n = 15)

Full-text articles assessed for eligibility
(n =20)

Records screened (title abstract)
(n =50)
