# Supplementary material for: The experiences of adjuvant endocrine therapy for women breast cancer survivors: A literature review
Source: Medicine (Baltimore). 2023 Dec 22;102(51):e36704. doi: 10.1097/MD.0000000000036704 (PMC10735117; doi:10.1097/MD.0000000000036704)
Supplement: Supplementary file 2 [file medi-102-e36704-s002.docx]

## Search Strategy

| Date | 4th Feb 2022 | |
| --- | --- | --- |
| Research Topic | The experiences of adjuvant endocrine therapy for women breast cancer survivors: a literature review | |
| Search Strategy | Key words/Concepts | Synonyms/Alternative terminology |
|  | Breast cancer | “Breast cancer” OR “breast cancer survivors” OR “breast cancer patients” OR “breast cancer clients” |
|  | Experiences | “Experiences” OR “perceptions” OR “attitudes” OR “views” OR “feelings” OR “perspective” OR “opinion” OR “qualitative” |
|  | Adjuvant endocrine therapy | “Adjuvant endocrine therapy” OR “endocrine therapy” OR “AET” OR “ET” OR “tamoxifen” OR “aromatase inhibitors” OR “drug therapy” OR “hormone therapy” |
| Limits and Type of material required | 2012 -2022 | |
|  | English language | |
|  | Journal article | |
|  | Peer reviewed | |
| Databases and Resources to be searched (incl. relevant organizations) | CINAHL | |
|  | MEDLINE/complete | |
|  | PsychArticles | |
|  | PubMed | |
|  | Cochrane Library | |
|  | Academic Search Complete | |
