# Supplementary material for: The experiences of adjuvant endocrine therapy for women breast cancer survivors: A literature review
Source: Medicine (Baltimore). 2023 Dec 22;102(51):e36704. doi: 10.1097/MD.0000000000036704 (PMC10735117; doi:10.1097/MD.0000000000036704)
Supplement: Supplementary file 3 [file medi-102-e36704-s003.docx]

## Table of Articles

| Author/ Study Title/Year | Research Aim | Methodology and Data collection | Sample | Major findings | Recommendations | Strengths and Limitations |
| --- | --- | --- | --- | --- | --- | --- |
| 1.Lambert, L.K., Balneaves, L.G., Howard, A.F., Chia, S.K. and Gotay, C.C. /Understanding adjuvant endocrine therapy persistence in breast Cancer survivors/2018 | To explore breast cancer survivors’ experiences and perspectives of AET use to describe how personal, social, and structural factors influence AET persistence. | Qualitative（An interpretive description methodology）,  Semi-structured interview | 22 participants,  Canada | 1.There was a growing body of evidence to support the impact of personal factors.  2. Survivors’ decision to persist with AET was a balancing act between QOL and quantity of life and was informed by a complex interplay of factors. The relative weight women attributed to QOL and quantity of life at different points in the AET trajectory was grounded in their personal experience and how social and structural factors influenced the broader context of their AET decisions and behaviors. | It was recommended that  further exploration of how the social and structural context in which AET decisions and behaviors are enacted is needed to guide the development of novel supportive care interventions. As well, it would be important to gain the perspectives of HCPs who support women undergoing AET to inform practical intervention strategies that can be implemented into routine clinical practice. | Strengths：  A relational autonomy lens was used to explore how personal, social, and structural factors shape women’s AET experiences.  Limitations：  1.The sample size was small and comprised of predominately well-educated Caucasian women who reported a high socioeconomic status.  2. It cannot to distinguish between these two categories of AETs.  3. It did not account for the experiences and perspectives of HCP.  4.May have experienced recall bias. |
| 2. van Londen, G.J., Donovan, H.S., Beckjord, E.B., Cardy, A.L., Bovbjerg, D.H., Davidson, N.E., Morse, J.Q., Switzer, G.E., Verdonck-de Leeuw, I.M. and Dew, M.A./ Perspectives of Postmenopausal Breast Cancer Survivors on Adjuvant Endocrine Therapy-Related Symptoms/2014 | To conduct an investigation of women's experiences related to taking AET and managing AET-related symptoms. | Qualitative,  A focus group design and  Semi-structured interview | 14 participants,  America | 1. Survivors on AET therapy encountered substantial challenges related to their experience and management of AET-related symptoms.  2.Although women were highly adherent despite unanticipated symptoms, women’s insights provided opportunities for patient and provider targeted interventions to improve AET-related symptom management, which could ultimately improve AET adherence, and survival. | It was recommended that further research should explore whether the AET experiences reflected in the current study are due to inadequate education from the provider or the survivor's ability to participate in and remember the conversation, and explore whether early, proactive, intermittent education about the benefits and expected risks of AET, as well as the assessment and management of AET-related symptoms, can improve survivors' ability to adhere to AET. | Strengths：  This study highlighted the important role that nurses can play in the ongoing assessment and management of AET-related symptoms.  Limitations：  1. Due to the sample reasons, the results of the study were not universal, such as most survivors in their 50s have jobs and therefore may have supporting resources that not all survivors have access to.  2. Focus on women who have been taking AET for at least one year. In these women, symptoms have not (at least) led to early termination of treatment. |
| 3.Wells, K.J., Pan, T.M., Vázquez-otero, C., Ung, D., Ustjanauskas, A.E., Muñoz, D., Laronga, C., Roetzheim, R.G., Goldenstein, M., Carrizosa, C., Nuhaily, S., Johnson, K., Norton, M., Sims, E. and Quinn, G.P. /Barriers and facilitators to endocrine therapy adherence among underserved hormone-receptor-positive breast cancer survivors: a qualitative study/2016 | To evaluate the barriers and facilitators to taking anti-hormonal medications among medically and historically underserved breast cancer survivors within the first five years post chemotherapy, radiation, and/or surgery. | Qualitative（A cross-section qualitative study）,  In-depth interview | 25 participants,  America | 1. Adherence to endocrine medications was promoted in many ways.  2. A small number of participants said they had difficulty paying for the drug.  3. Side-effects were the most mentioned drug adherence disorders. A small number of participants reported that they experienced psychological side-effects due to the drug.  4. They believed that it was important to get additional information about the side-effects of the drug, which will help them decide which drug to take and what side effects to expect. | It was recommended:  1.At the time an anti-hormonal medication was prescribed, survivors should know comprehensive information regarding the side-effects associated with the prescribed medication and the likelihood of these side effects occurring. Information should also be presented regarding the risk of breast cancer recurrence associated with not taking the medication.  2. Ongoing assessment and management of side-effects should be necessary.  3.Future studies should engage HR+ breast cancer survivors who become lost to follow-up post chemotherapy, radiation, and surgery to determine if they experience other barriers to care not reported in the present study. | Strengths：  These findings contributed to the scientific literature regarding adherence to anti-hormonal medications and would be used to develop a patient navigation intervention to improve receipt of breast cancer survivorship care, including adherence to anti-hormonal medications.  Limitations：  1. Participants were recruited from one comprehensive cancer center.  2. Participants were still receiving health care at the comprehensive cancer center.  3.The findings may not generalize to patients from other areas of the US or from other countries, as well as to breast cancer survivors who were either not receiving survivorship care or who were receiving survivorship care outside of a comprehensive cancer center. |
| 4.Hurtado-de-Mendoza, A., Jensen, R.E., Jennings, Y. and Sheppard, V.B./Understanding Breast Cancer Survivors’ Beliefs and Concerns about Adjuvant Hormonal Therapy: Promoting Adherence/2018 | To explore the clarity and relevance of the BMQ in the relation to hormonal therapy in a racially diverse sample of Black and White breast cancer survivors. | Qualitative（A qualitative descriptive study) ,  Cognitive interview and in-depth interview | 30 participants,  America | 1. There were no racial differences in relevance and clarity of BMQs.  2.Two white survivors who stopped the drug early identified side-effects and effects on quality of life as the main causes.  3. Participants expressed ambivalence, they knew that hormone therapy could improve survival, but also had difficulty dealing with side effects and how they affected their daily lives. | It was recommended:  1.Future studies should capture relevant beliefs and ambivalent attitudes.  2. Future larger studies should examine whether race and other socio-demographic factors impact the perceptions of relevance and clarity of the BMQ.  3. Future studies should explore further survivor’s experiences taking hormonal therapy.  4. Adjuvant hormonal therapy should be recommended for women with hormone receptor positive (HR) breast cancer. | Strengths：  1. This was the first descriptive study to examine the clarity and relevance of BMQ in the context of hormonal therapy adherence in breast cancer survivors.  2.The results of this study could provide insights into the development of new instrument.  Limitations：  1. The sample was a small convenience sample of women with a high level of education.  2. Two participants spontaneously reported that they had discontinued treatment, but the study did not explicitly ask about discontinuation.  3.It cannot know how many women had actually stopped taking the medication. |
| 5.Jacobs, J.M., Walsh, E.A., Park, E.R., Berger, J., Peppercorn, J., Partridge, A., Horick, N., Safren, S.A., Temel, J.S. and Greer, J.A./The Patient’s Voice: Adherence, Symptoms, and Distress Related to Adjuvant Endocrine Therapy After Breast Cancer/2020 | To understand patient experiences on AET, motivators and barriers to adherence, side effects, and distress, with the goal of developing a patient-centered, evidence-based intervention. | Qualitative,  In-depth semi-structured interview | 30 participants,  America | 1. The themes highlighted the experiences taking AET that could serve as modifiable targets and inform a patient-centered intervention.  2. Survivors experienced challenges related to AET.  3.Although mostly adherent to AET and motivated to reduce risk of recurrence, patients described strong ambivalence, negative beliefs, and concerns regarding AET.  4. Those with low adherence endorsed greater challenges with side-effects, more ambivalence towards AET, and more difficulties with mood.  5. Survivors expressed a preference for a group-based intervention. | It was recommended that evidence-based interventions for adherence, symptom management, and pain should be recommended, combining patient preference for AET with social support for others. | Strengths：  1. This study examined individual preferences for a psychosocial intervention content and logistics in order to maximize feasibility, acceptability, and eventual efficacy.  2. The study explored differences in qualitative themes by patient characteristics, such as age, distress level, and the amount of time on AET.  Limitations：  1. The study purposefully recruited a select number of patients who reported low or high adherence and only high adherers with moderate to severe side effects. |
| 6.Moon, Z., Moss ‐ Morris, R., Hunter, M.S., Hughes, L.D. and Moss-Morris, R./ Understanding tamoxifen adherence in women with breast cancer: A qualitative study/2017 | To understand women’s experiences of taking tamoxifen and to identify factors which may be associated with non-adherence. | Qualitative,  Semi-structured interview | 32 participants,  England | 1.The main reason women were non-adherent or non-persistent with tamoxifen was because they were struggling with the side-effects, and they did not believe that the benefits of the treatment outweigh the side-effects. 2.Women expressed a need for more information about tamoxifen and side-effects information.  3. 23 women were classed as adherent, 4 were non-adherent, and 5 had discontinued. Two of the women discontinued on their doctors’ recommendations.  4. Adherence rates may be related more to the perceived impact of side effects than the side effects themselves. | It was recommended:  1.Future research needs to explore ways to increase beliefs around tamoxifen necessity and how to help women cope with side effects.  2. Women should be given personalized information, so they could make decisions about tamoxifen based on the extent to which it would benefit them. Women also wanted to be warned about what side-effects to expect.  Patients also needed to be informed about the importance of taking tamoxifen as prescribed. | Strengths:  1.These results provided insight into the experiences of patients who initiated tamoxifen.  2.This study contributed new understanding by moving beyond the generic model and showing the specific beliefs held by these patients and how they influenced behavior.  Limitations:  1. Women who had chosen not to initiate tamoxifen were not included in the study  2. It may have under-represented women who were non-adherent as there may be a selection bias.  3. There may be issues of recall bias. |
| 7.Humphries, B., Collins, S., Guillaumie, L., Lemieux, J., Dionne, A., Provencher, L., Moisan, J. and Lauzier, S./ Women’s Beliefs on Early Adherence to Adjuvant Endocrine Therapy for Breast Cancer: A Theory-Based Qualitative Study to Guide the Development of Community Pharmacist Interventions/2018 | To identify women’s attitudinal, normative, and control beliefs regarding AET adherence that could be targeted by an intervention offered in the community pharmacy setting. | Qualitative（A qualitative descriptive study  ）,  Focus groups and individual interviews | 43 participants,  Canada | 1. Most women had a positive attitude towards AET regardless of their medication-taking behavior.  2. The principal advantage perceived by participants was protection against a recurrence while the principal inconvenience was side effects.  3.Women particularly valued the support of the health care team, their relatives, and cancer survivors. Interventions for the community pharmacy setting should therefore target these three sources of social support.  4. For adherent women, having trouble establishing a routine was the main barrier to taking their medication. For non-adherent women, it was side-effects and a diminished quality of life. | It was recommended:  1. Community pharmacists may need additional training regarding AET counselling and monitoring.  2. It may be challenging for community pharmacists to perform drug monitoring throughout the 5 or 10 years of AET treatment. An assessment of community pharmacists’ ability, needs and attitudes toward performing these interventions should be conducted to complement results from the present study. | Strengths:  1. It was the first qualitative study to use the TPB to explore the experiences of women with an AET prescription. The use of the TPB contributed to our understanding of beliefs that should be targeted in a community pharmacy-based intervention aimed at enhancing AET adherence.  Limitations:  1. Sample was restricted to women prescribed AET within the last two years. However, medication-taking behaviors can change over time.  2. It was generally more difficult to recruit patients with non-adherent behaviors because they were less inclined to share their experience. |
| 8.Wickersham, K., Happ, M.B. and Bender, C.M./ “Keeping the Boogie Man Away”: Medication Self-Management among Women Receiving Anastrozole Therapy/2012 | To describe the medication-taking experiences for postmenopausal women with early-stage breast cancer who were prescribed a course of anastrozole therapy. | Qualitative（A qualitative descriptive study  ）,  Semi-structured interview | 12 participants,  America | 1. Women in the current study had six-month adherence levels ranging from 38.4% to 100% (mean = 87.8%).  2. Participants reported three to six side-effects and most women reported five or more.  3. Though their side-effect experiences were significant, the women remained motivated to take anastrozole; only one woman stopped taking anastrozole due to side-effects.  4.All medication-taking practices were facilitated by routinization.  5. Women who took anastrozole without question believe in the medication's value and importance.  6.Women received little in the way of instructions concerning medication use, side-effects, and daily management of anastrozole. | It was recommended:  1.Provision of information about anastrozole, its side -effects, and how and when to take it may be beneficial, beginning with the first clinic visit with ongoing reassessment at subsequent clinic visits.  2. Focusing on the patient's medication-taking experiences as a whole.  3. Next steps should include investigations of medication-taking about different breast cancer survivors. | Strengths：  It offered a unique perspective into the medication-taking experiences of some postmenopausal women with early-stage breast cancer.  Limitations：  1. The most significant limitation in the current study was the potential influence of participation in The AIM Study. It may have affected survivor’s responses.  2. All women were white and well educated.  3. Sampling strategies did not provide a comprehensive picture of how women took anastrozole.  4. It did not saturate with regard to those who were low-adherers or who had stopped AI therapy. |
| 9. Paulo, T.R.S., Rossi, F.E., Viezel, J., Tosello, G.T., Seidinger, S.C., Simões, R.R., de Freitas, R. and Freitas, I.F./The impact of an exercise program on quality of life in older breast cancer survivors undergoing aromatase inhibitor therapy: a randomized controlled trial/2019 | To evaluate the impact of an exercise program on quality of life in older breast cancer survivors undergoing aromatase inhibitor therapy. | Quantitative  (RCT),  Questionnaire | 36 participants（18werecontrol group and 18 were intervention group ）,  Brazil | 1. Combined resistance and aerobic training can improve physical, psychological, and social functioning outcomes, with potential benefits for quality of life and health in older breast cancer survivors treated with AI, also contributing to public health actions.  2. Higher adherence for the exercise group.  3. Older breast cancer survivors can adapt to an exercise program.  4.Fatigue, sleep disturbance and bodily pain can be improved with a combined exercise program. | It was recommended that implementing a combination of aerobic and resistance training may be an important strategy to improve health and minimize the effectiveness of breast cancer treatment. | Strengths：  This study demonstrated the potential benefits and high clinical relevance of exercise programs to improve quality of life in older breast cancer survivors undergoing aromatase inhibitor therapy.  Limitations：  1.The sample size was small.  2.The lack of performance tests for measuring physical function.  3. Self-reported assessments of questionnaires, which were generally inferior to objective measures. |
| 10. Cahir, C., Dombrowski, S.U., Kelly, C.M., Kennedy, M.J., Bennett, K. and Sharp, L./Women’s experiences of hormonal therapy for breast cancer: exploring influences on medication-taking behaviour/2015 | To investigate modifiable influences on adjuvant hormonal therapy medication-taking behaviour (MTB) in women with stage I-III breast cancer. | Qualitative,  Semi-structured interview | 31 participants,  Ireland | 1.Adherent and persistent women strongly believed in the necessity and efficacy of their treatment, were highly motivated and adapted a wide range of coping techniques and support networks to enable them to take their treatment.  2.Non-adherence was associated with inadequate medication management techniques, a general distrust in medication, concerns and anxiety over treatment side effects and lack of knowledge and conviction in the efficacy of the treatment.  3.Non-persistence was associated with a strong distrust of medication and the health care system, a lack of perceived need for treatment and a preference for a good quality of life with little concern or thought given to future outcomes. | It was recommended that targeting these influences in clinical practice may improve MTB and hence survival in this population. | Strengths：  1. This was the first study to apply the TDF to hormonal therapy MTB and used the TDF to identify a wide range of potential influences, and study was not confined to a particular psychological theory or groups of determinants.  Limitations：  1.Although women were recruited from two cancer centers, there was a diversity of opinions expressed, and participants were diverse in socio-demographic characteristics as well as their MTB; So, it was likely that the generalizability of themes was high.  2. There was a critical lack of understanding as to why women took or did not take their hormonal therapy. |
| 11.Farias, A., Ornelas, I., Hohl, S., Zeliadt, S., Hansen, R., Li, C., Thompson, B., Farias, A.J., Ornelas, I.J., Hohl, S.D., Zeliadt, S.B., Hansen, R.N. and Li, C.I./ Exploring the role of physician communication about adjuvant endocrine therapy among breast cancer patients on active treatment: a qualitative analysis/2017 | To better understand how physicians communicate with breast cancer patients about adjuvant endocrine therapy (AET). | Qualitative,  Semi-structured in-depth interview | 22 participants,  America | 1. Four major functions of physician-patient communication: (1) information exchange, (2) decision-making to take and continue AET, (3) enabling patient self-management and monitoring potential side effects, and (4) emotional support.  2. Patients reported a high degree of self-efficacy to self-manage AET and were continuing treatment despite potential side effects.  3. physician-patient communication plays an important role in both the initiation and the long-term continued use of AET for women with breast cancer. | It was recommended:  1.Women should interact and communicate with their physician.  2. Physicians who can communicate information about AET in a way that patients can understand was a critical aspect of care that needed to be further studied.  3. Future research should explore the influence of the social and cultural environment and how these relationships support the use of AET.  4. Future research should examine the physician-patient interactions of patients who discontinue or never initiate AET treatment and whether or not interactions differ by race/ethnicity. | Strengths:  The study provided insight into the physician-patient interactions that occur among women actively taking AET and identified important functions of physician communication that could be addressed in order to improve the use of AET.  2.The findings were supported by a theoretical framework.  Limitations:  1. Participants were highly educated and had an income at or above the US median household income, so the study did not consider the cost of AET medication. |
| 12.Harrow, A., Dryden, R., McCowan, C., Radley, A., Parsons, M., Thompson, A.M. and Wells, M./A hard pill to swallow: a qualitative study of women’s experiences of adjuvant endocrine therapy for breast cancer/2014 | To explore women's experiences of taking adjuvant endocrine therapy as a treatment for breast cancer and how their beliefs about the purpose of the medication, side effects experienced and interactions with health professionals might influence adherence. | Qualitative,  Semi-structured interview | 30 participants,  England | 1.Women sought to be adherent, but some would miss tablets without realizing the potential consequences.  2.The impact of severe side-effects did not necessarily affect adherence, as women believed that taking the medication reduced their risk of recurrence outweighs these negative effects.  3.Not all women who experience side effects will seek advice and support.  4. 5 out of thirty women described missing one or two tablets now and then, 9 women had stopped temporarily (n=6) or permanently (n=3), either following clinical advice or of their own volition. The remaining 16 women had taken their medication every day. | It was recommended:  1.Providing follow-up settings. Women should deserve more opportunity to discuss the pros, cons and impact of long-term AET.  2.New service models should be needed to support adherence, enhance quality of life and ultimately improve survival. These should ideally be community based, in order to promote self-management in the longer term.  3.A patient-centered approach was likely to facilitate adherence. | Strengths:  This was one of the few studies which have asked women to talk about their experiences of taking AET for breast cancer.  Limitations:  1. Women with low adherence and those who were premenopausal were under-represented.  2. This study was conducted in only two centers with the majority of participants recruited from a single center. |
| 13. Liu, Y., Malin, J.L., Diamant, A.L., Thind, A. and Maly, R.C./ Adherence to adjuvant hormone therapy in low-income women with breast cancer: the role of provider-patient communication/2013 | To assess the impact of patient-provider communication on adherence to tamoxifen (TAM) and aromatase inhibitors (AI) 36 months after breast cancer (BC) diagnosis in a low-income population of women. | Quantitative (An observational  study  ) ,  Questionnaire | 921 participants,  America | 1.Overall adherence to AET was relatively high (88%).  2.Adjusted rates of adherence were 59% and 94% for patients with the lowest vs highest scores on the CAHPS communication scale and 72% vs. 91% for patients with the lowest and highest rating of PEPPI.  3.Having no health insurance and experiencing side-effects from hormone treatment were barriers for adherence.  4. Patient-centered communication and perceived self-efficacy in patient-physician interaction were significantly associated with patient adherence to ongoing AET among low-income women with survivors.  5. Less acculturated Latinas were significantly more likely than whites to remain on hormone therapy 3 years after the breast cancer diagnosis. | It was recommended:  1.Interventions on patient-provider communication should be provided.  2. Further research should be needed to explore the contributing factors to the ethnic variation in adherence found in this study. | Strengths:  Its results suggested that adherence to hormone therapy among low-income, medically underserved women with survivors could be enhanced by targeted interventions aimed at increasing patient-centered communication and by attention to side effects from the treatment.  Limitations:  1. The sample was comprised of low-income, medically underserved women.  2.Although the study achieved a 61% response rate, the results might potentially have biased.  3. Since adherence was measured by self-report, the high adherence rate may have been affected by social desirability response bias. |
| 14.Moon, Z., Moss-Morris, R., Hunter, M.S., Norton, S. and Hughes, L.D./ Nonadherence to tamoxifen in breast cancer survivors: A 12 month longitudinal analysis/2019 | To examine how tamoxifen adherence rates change across a 1-year period, and to identify modifiable predictors of nonadherence, using two social cognition models of health behavior as a framework. | Quantitative （A longitudinal study） ,  Questionnaire | 345 participants,  England | 1.Reported rates of nonadherence increased over time (37–48%).  2. Young, ethnic minority groups, employed and higher levels of distress，women may be at higher risk of nonadherence.  3. Demographic factors, such as ethnicity, age and employment status were associated with unintentional nonadherence, whereas psychological factors such as perceptions around risk of recurrence tended to be associated more with intentional nonadherence.  4.Self-reported side-effect intensity increased significantly over the 12-month period.  5. Clinicians could identify these women and give them additional support with their medication taking and with managing distress. | It was recommended:  1.These demographic and clinical variables can be used to identify women at higher risk of nonadherence.  2.The modifiable psychosocial variables can be used as the basis for psychological interventions to improve adherence in this population.  3.Interventions should focus on both intentional and unintentional nonadherence.  4. Future research should explore the relationship between ethnicity and nonadherence.  5. Future research could extend this to women prescribed aromatase inhibitors. | Strengths：  1. This study was one of the first to identify modifiable psychosocial predictors of nonadherence to tamoxifen longitudinally.  2.These results provided important information on how to support women from ethnic minorities taking tamoxifen.  Limitations：  1. While retention rates were relatively high, significant differences were seen between responders and no responders. Women who did not respond were more likely to be younger, from a minority ethnic group and to be more nonadherent at baseline.  2. There was little ethnic diversity in the sample.  3. The research only focused on women prescribed tamoxifen. |
| 15. Graetz, I., McKillop, C.N., Stepanski, E., Vidal, G.A., Anderson, J.N. and Schwartzberg, L.S./Use of a web-based app to improve breast cancer symptom management and adherence for aromatase inhibitors: a randomized controlled feasibility trial/2018 | To evaluate the feasibility and short-term effect of use of a web-based communication app designed for breast cancer patients to report adverse symptoms and AI adherence outside of clinic visits, with builtin alerts sent to patients’ care teams | Quantitative  (A randomized controlled feasibility trial  ),  Questionnaire | 44 participants（23werecontrol group and 21 were intervention group ）,  America | 1.Participants in the intervention group had higher weekly app usage rate (74% vs. 38%, p<0.05) during the intervention and reported higher AI adherence at 8 weeks (100% vs 72%, p<0.05).  2.Symptom burden increase was higher for the control group compared to the intervention group but did not reach statistical significance.  3. Patients and providers who participated in the trial conveyed a positive experience with the study app, citing that it was beneficial to providing and receiving care. | It was recommended:  1. The high penetration of web-enabled devices, including smartphones, could be leveraged by the healthcare community as a way to further engage patients between visits in order to improve care quality and health outcomes.  2.Future studies should test if short-term gains in AI adherence can be maintained over the full treatment period (typically 5–10 years), and if improved adherence is associated with clinically meaningful improvements in health outcomes. | Strengths：  Weekly reminders to use a web-based app to report AI adherence and treatment-related symptoms demonstrated feasibility and improved short-term AI adherence, which may reduce symptom burden for women with breast cancer and a new AI prescription.  Limitations：  1. it was not powered to detect statistically significant differences in the study outcomes.  2. The study only followed participants for up to eight weeks.  3. Using self-reports had been found to be less reliable than electronic monitoring systems or pill counts.  4. The study recruited patients from a single clinic. |

AET=Adjuvant Endocrine Therapy, AIs=Aromatase Inhibitors, MTB=Medication-Taking Behavior, HCP= Healthcare Professionals
